# Unravelling the Diversity of Grapevine Microbiome

**DOI:** 10.1371/journal.pone.0085622

**Published:** 2014-01-16

**Authors:** Cátia Pinto, Diogo Pinho, Susana Sousa, Miguel Pinheiro, Conceição Egas, Ana C. Gomes

**Affiliations:** 1 Genomics Unit, Biocant - Biotechnology Innovation Center, Cantanhede, Portugal; 2 Advanced Services Unit, Biocant - Biotechnology Innovation Center, Cantanhede, Portugal; Loyola University Medical Center, United States of America

## Abstract

*Vitis vinifera* is one of the most widely cultivated fruit crops with a great economic impact on the global industry. As a plant, it is naturally colonised by a wide variety of both prokaryotic and eukaryotic microorganisms that interact with grapevine, having either beneficial or phytopathogenic effects, who play a major role in fruit yield, grape quality and, ultimately, in the evolution of grape fermentation and wine production. Therefore, the objective of this study was to extensively characterize the natural microbiome of grapevine. Considering that the majority of microorganisms are uncultivable, we have deeply studied the microflora of grapevine leaves using massive parallel rDNA sequencing, along its vegetative cycle. Among eukaryotic population the most abundant microorganisms belonged to the early diverging fungi lineages and Ascomycota phylum, whereas the Basidiomycota were the least abundant. Regarding prokaryotes, a high diversity of Proteobacteria, Firmicutes and Actinobacteria was unveiled. Indeed, the microbial communities present in the vineyard during its vegetative cycle were shown to be highly structured and dynamic. In all cases, the major abundant microorganisms were the yeast-like fungus *Aureobasidium* and the prokaryotic Enterobacteriaceae. Herein, we report the first complete microbiome landscape of the vineyard, through a metagenomic approach, and highlight the analysis of the microbial interactions within the vineyard and its importance for the equilibrium of the microecosystem of grapevines.

## Introduction

*Vitis vinifera* naturally hosts a reservoir of microorganisms that interact with it and can be transferred to grapes and then into the winery where, ultimately, it may affect the wine production and compromise its quality. Those microorganisms can be beneficial, neutral or pathogenic to the plant [1], [2].

The grapevine is also colonized by other ubiquitous microorganisms known as epiphytes and endophytes, who could have beneficial or neutral effects on plants, without causing disease symptoms [3]. Some of these microorganisms are even considered as natural biocontrol agents due to their ability to protect the plant against phytopathogens and reinforce the natural plant defences [4].

Indeed, grapevine is susceptible to several phytopathogens attacks with negative impact on vine longevity, affecting the plant vitality [5]. Altogether, they compromise the normal physiological properties of the grapevine and its vigour, resulting in a decrease of fruit yield and quality, and thus undermining the expected economic return of the vineyard. The most critical phytopathogens agents are fungi, viruses and phytoplasmas [6], [7].

The balance of the grapevine microecosystem is influenced by biotic and abiotic factors and also by spatial and temporal fluctuations. In addition, the chemical treatments also affect the grapevine microbiome and are responsible for the appearance of pesticide-resistant pathogen strains [8]. Therefore, a complete survey of the grapevine's microbial ecology, under natural conditions, is of outmost importance because the grape production and quality can be affected by the vineyard's active microbial community [9].

Indeed, phytopathogens have a direct negative impact on grapevine and cause blighting, shrivelling, vine decay and tissue damage [10]. Moreover, the microbial secondary metabolites as mycotoxins, produced by some moulds, are toxic metabolites that may later contaminate the wines. An example of a relevant mycotoxin present in wines, with highest impact in red wines, is the ochratoxin_A (OTA) that is produced by *Aspergillus* spp. and *Penicillium* spp. [11], [12]. On the other hand, the microbial community can activate the plant defence pathways, inducing the accumulation of pathogenesis-related (PR) proteins of grapevine as a protection against fungal pathogen attacks or other biological stresses [5]. In fact, it is well known that the accumulation of such PR proteins as chitinases and taumatin-like proteins will later affect the wine clarity and stability [5], [13].

Altogether, microorganisms are important for the equilibrium of ecosystems, although little is known about the magnitude and variability of those populations under natural conditions [14]. Indeed, the majority of studies characterizing the microbial diversity rely on classical microbiological approaches. However, cultivation-independent molecular techniques are now starting to be widened, and metagenomics, the study of all indigenous biota from samples, represents a powerful tool for assessing the microbial communities [15]–[17].

In our study we have used 454 Next-Generation Sequencing (NGS) to sequence the rDNAs of all microorganisms present in the vine's samples. With these data we will be able to identify both abundant and rare microorganisms present on the vineyard and therefore unravel the dynamics of microbial population during the grapevine vegetative cycle.

## Materials and methods

### Sampling procedures and DNA extraction

A vineyard with 10 ha located in Bairrada appellation (Cantanhede, Portugal) was chosen for this study (Figure S1a). The sampling was authorized by the private owner, who is fully acknowledged in this paper, and no specific permissions were required for this activity. Also, the field study did not involve endangered or protected species. In order to obtain the deepest insight on the microbial biodiversity, we have collected both healthy (asymptomatic) and diseased leaves from *V. vinifera* cv Tempranillo (also known as Aragonez and Tinta Roriz). For this study, we have decided to sample leaves as they are the biomarkers for the phytosanitary status of plant, are the most abundant, and are the organ of highest surface of the plant. Comparatively to other structures as fruits or flowers, which are not always present, the leaves are the most permanent structures, thus allowing the study along the vegetative cycle of the plant. Furthermore, leaves are more convenient to sample than the wood, which would require cuttings and thus would jeopardize the vitality of the vine. The leaf samples were repeatedly collected during the vegetative cycle from May to July in a total of 10 samplings, from T1 to T10. Sampling was done in 5 different vines distributed in the vineyard, before and after chemical treatments (Figure S1b). The sampling was carried out always from the same vines all over the experiment, in order to minimize sources of variability within this study. A total of 50 leaves were collected and stored at −80°C for subsequent DNA extraction. The DNA, from individual grapevine leaf samples, was extracted using the QIAamp® DNA Stool Mini Kit (Qiagen, Hilden, Germany), according to the manufacturer's instructions.

### Amplification of prokaryotic and eukaryotic population and pyrosequencing

A PCR amplicon library was built with the extracted DNA. The PCR primers were designed to target the V6 region of the 16S rDNA for prokaryotic population analysis and the ITS2 and D2 rDNA sequences for eukaryotic population study. A preliminary analysis of our results demonstrated that species obtained with D2 and ITS2 region sequencing are different and that the common taxonomic organisms are low (Figure S2). Therefore, here in we have sequenced and analysed both regions to have the most complete landscape of the vineyards microbiome.

Distinct PCR reactions were performed for V6, D2 and ITS2 region. The amplification of the PCR products was carried out in a 30 µL reaction mix containing 1× reaction buffer (USB, Affymetrix), 0.2 mM of MgCl_2_ (USB, Affymetrix), 0.2 mM dNTPs (Bioron), 2 µL of DNA, 1 unit of FideliTaq DNA Polymerase (USB, Affymetrix) and 0.4 µM of the eukaryotic forward and reverse specific primer or 0.8 µM of prokaryotic primers. Both eukaryotic and prokaryotic regions were amplified with primers containing the 454 Life Science's sequence adaptors (5′-CGTATCGCCTCCCTCGCGCCATCAG-3′), a barcode with 8 nucleotides which allowed the pooling of multiple samples for pyrosequencing, and the universal primers. The ITS2 region was amplified with the primers ITS2_F 5′-GCATCGATGAAGAACGC-3′ and ITS2_R 5-‘CCTCC GCTTATTGATATGC-3’, the D2 region was amplified with D2_F 5′AAGMACTTTGRAAAGAGAG-3′ and D2_R 5′-GGTCCGTGTTTCAAGACG-3′ and the V6 region with the primers V6_F 5′-ATGCAACGCGAAGAACCT-3′ and V6_R 5′-TA GCGATTCCG ACTTCA-3′. Cycling conditions consisted of an initial denaturation step at 94°C for 5 min; followed by 25 cycles at 94°C 35 s, 50°C 35 s and 68°C for 40 s; and a final extension for 5 min at 68°C. The PCR amplicons were analysed with the HT DNA 5000 SE30 Chip for the LabChip 90 (Caliper LifeSciences, USA). The PCR products were purified with the High Pure 96 UF Cleanup Plates (Roche) and then their quality and quantity were assessed by fluorimetry, using the PicoGreen® dsDNA quantitation kit (Invitrogen, USA). Afterwards, samples were pooled together in equimolar amounts, and the fragments in the amplicon library were bound to beads under conditions that favour one fragment per bead. The fragments in the amplicon library were subject to an emulsion PCR and the resulting DNA library beads were deposited into the PicoTiterPlate (PTP) for high-throughput pyrosequencing using the Genome Sequencer FLX System Instrument (454 Life Sciences, Roche) at Biocant, Portugal. All sequences obtained from this work are publically available in NCBI platform with the accession number SRP029989.

### Data analysis

The raw data was analysed by an automatic annotation pipeline implemented at the Bioinformatics Unit of Biocant. The sequence reads obtained were sorted by identification TAGs and quality filters were applied in order to remove low-quality reads. We have eliminated (*i*) sequences with less than 120 pb, (*ii*) sequences that contained unresolved nucleotides (>2), (*iii*) masked sequences with more than 50% of low complexity areas [18], (*iv*) chimera sequences detected using UChime [19]. Sequences were then grouped according to their phylogenetic distance of 3% [14] and grouped in Operational Taxonomic Units (OTU) through USearch [20]. The consensus sequences were automatically obtained by this software. These pairwise distances served as input to Mothur package [21] for the generation of rarefaction curves (richness of population analysis) and the calculation of the population diversity analysis estimator Chao1 (α diversity). Consensus sequences for each OTU were blasted against curate databases which allowed for taxonomic annotation. Prokaryotic microorganisms were searched on Ribosomal Database Project II (RDP) database [22], whereas eukaryotic microorganisms were identified on the nt@ncbi/SILVA database. After BLAST, the best hits were selected and subjected to another quality control: only the sequences with an alignment greater than 60% and an e-value lower than 1e^−5^ were selected and applied for a bootstrap test with 100 replicates, which were obtained by seqBoot from Phylip package [23]. Only those sequences with an identity greater than 70% were accepted, while all the others were considered new sequences.

Eukaryotic and prokaryotic data were analyzed to determine the minimum significant difference (ρ<0.05) between biodiversity (Chao1) and one-way analysis of variance (ANOVA) was performed by employing SPSS 20.0 (IBM, US). Normality tests (Shapiro-Wilk) were carried out for each month of collections (May, June, July) and for interval betweenchemical treatments. As all groups followed the normal distribution, a T-test was used.

The microbial communities present from T1 to T10 were compared at family level for prokaryotic microorganisms and at genus level for eukaryotic population through the sequence reads analysis. Thus, microbial population comparisons were carried out using these taxa. Nevertheless, in some cases it was possible to achieve a sound identification of the species sequence (table S1), mostly for the eukaryotic population, which are also herein discussed.

To analyze the community composition, a log-transformed (log _10_(x+1)) of microbial community was performed. Then, to compare the microbial community structure across the different times of collection (T1–T10), a Principal Component Analysis (PCA) was performed with Bionumerics 6.5 (Applied Maths NV, Belgium). The scores and loadings values were exported and both plots were designed in Excel 2010 (Microsoft, USA). Metastats [24] was used to detect differentially abundant taxa in two microbial populations (microbial population before and after chemical control and during vegetative cycle – May, June, July) and to assess the significance of the observed differences in microbial community. The heat maps were done using Bionumerics 6.5 (Applied Maths NV, Belgium).

## Results

### Microbial population diversity and richness of grapevines

The objective of this work was to assess the microbial community from grapevine leaves, during the vineyard's vegetative cycle, using a culture independent approach. To achieve this we have undergone a DNA massive parallel sequencing of 16S rRNA gene and D2 and ITS2. Throughout the vegetative cycle of the grapevine, a total of 50 leaf samples were collected from V. vinifera cv Tempranillo and samples were collected before and after the application of chemical treatment according to the calendar of Figure S1b. The deep sequencing of microbial communities originated a total of 142 096 sequences, of which 139 034 sequences passed the Quality Control filters, which represented 97.9% of the obtained sequences (Table 1). For eukaryotic microorganisms we have obtained 79 398 sequences (38 187 identified with D2 region and 41 211 with ITS2) and for prokaryotic we have obtained 59 636 sequences (Table 1). The number of reads per sample ranged from 2070 to 9462 sequences. All the high-quality sequence reads were grouped at a genetic distance of 3% and generated a total of 1 043 OTUs for ITS2, 895 for D2 and 1 242 for V6. On average, we have obtained 97±11 and 124±7 OTUs for eukaryotic and prokaryotic microorganisms, respectively.

**Table 1:** Total sequences obtained for eukaryotic (ITS2 and D2) and prokaryotic (V6) microbial community for all samples (T1–T10).

					0.03 distance			
Time Points	Target region	Total reads	High quality	OTU obtained	OTU that passed the blast	CHAO 1	ACE	Coverage (%)
**T1**	D2	3310	3284	118	111	173	171.37	64.2%
	ITS2	6958	6858	187	176	244	233.70	72.1%
	V6	9528	9462	91	88	127	125.49	69.4%
**T2**	D2	3371	3342	175	170	272	373.87	62.4%
	ITS2	5630	5454	244	228	392	518.71	58.1%
	V6	8258	8197	130	127	219	327.75	58.1%
**T3**	D2	3544	3511	109	99	212	208.34	46.6%
	ITS2	3176	3034	148	138	254	353.58	54.4%
	V6	6254	6127	134	126	233	355.03	54.1%
**T4**	D2	6758	6680	91	85	250	379.68	33.9%
	ITS2	4117	3828	91	84	186	280.17	45.2%
	V6	5534	5464	111	107	152	183.03	70.3%
**T5**	D2	4094	4071	72	69	116	116.77	59.5%
	ITS2	2765	2648	48	46	106	369.32	43.4%
	V6	6728	6627	169	163	242	254.01	67.2%
**T6**	D2	3902	3872	109	106	199	340.45	53.3%
	ITS2	3354	3197	121	115	245	358.78	46.9%
	V6	5763	5723	132	128	225	297.95	56.9%
**T7**	D2	2096	2070	48	47	87	123.31	54.3%
	ITS2	3712	3485	61	58	106	213.35	54.7%
	V6	3923	3872	104	103	176	206.29	58.6%
**T8**	D2	3325	3304	83	78	165	207.70	47.3%
	ITS2	3751	3576	74	73	110	109.81	66.5%
	V6	4383	4274	149	142	268	386.75	52.9%
**T9**	D2	4543	4487	72	68	146	192.89	46.5%
	ITS2	4518	4292	65	65	106	138.57	61.6%
	V6	5615	5553	157	146	250	304.77	58.3%
**T10**	D2	3612	3566	67	62	89	100.74	69.5%
	ITS2	5197	4839	61	60	127	186.99	47.2%
	V6	4377	4337	116	112	138	144.16	81.4%
**Total**	**D2**	38555	38187	944	895	1710	2215.11	52.3%
	**ITS2**	43178	41211	1100	1043	1875	2762.97	55.6%
	**V6**	60363	59636	1293	1242	2030	2585.22	61.2%
	**Eukaryotic**	81733	79398	2044	1938	3585	4978	54.4±2.2%
	**Prokaryotic**	60363	59636	1293	1242	2030	2585	62.7±2.7%
	**TOTAL**	**142096**	**139034**	**3337**	**3180**	**5616**	**7563**	

OTUs and estimated species (Chao1) were determined at a genetic distance of 3% using Mothur. The coverage obtained was also determined as being the ratio between the observed OTUs and the estimated Chao1 (OTUs/Chao1).

**Figure 1:**
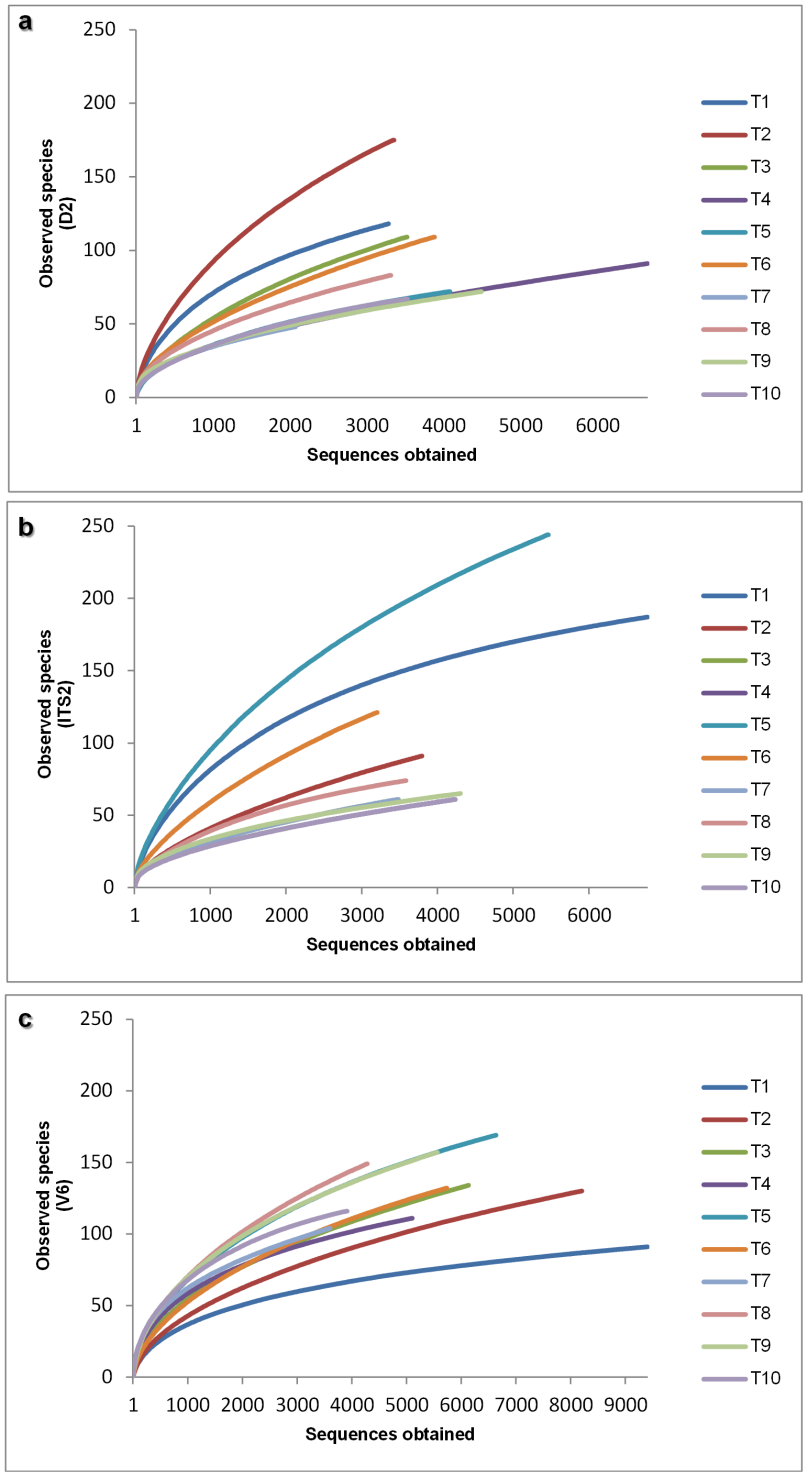
Rarefaction curves at a genetic distance of 3% for each sample (T1–T10). D2 (a) and ITS2 (b) sequences both from the analysis of 26S rRNA and ITS regions of eukaryotic population present in the sample and V6 sequences (c) from the analysis of 16S rRNA of prokaryotic diversity.

The diversity of eukaryotic and prokaryotic populations was compared between samples by rarefaction curves analysis (Figure 1). This allowed us to measure the deepness of our experiments and to characterize the microbial community [25]. Rarefaction curves showed that a good coverage of the entire community was achieved. Therefore, we are aware that despite unveiling a complex and rich microbial structure, there still exists a hidden biodiversity within the vineyard, which we were not able to expose (Table 1).

For each sample we have determined its expected richness (Chao1 index). In our analysis, we have predicted a total richness ranging from 179±17 (eukaryotic microorganisms) to 203±15 (prokaryotic population). By comparing the obtained number of OTUs with its predicted Chao1, we were able to determine the coverage of our experiments. The richness estimators indicated that 54.4±2.2 % and 62.7±2.7 % of the eukaryotic and prokaryotic diversity was uncovered, respectively (Table 1).

**Figure 2:**
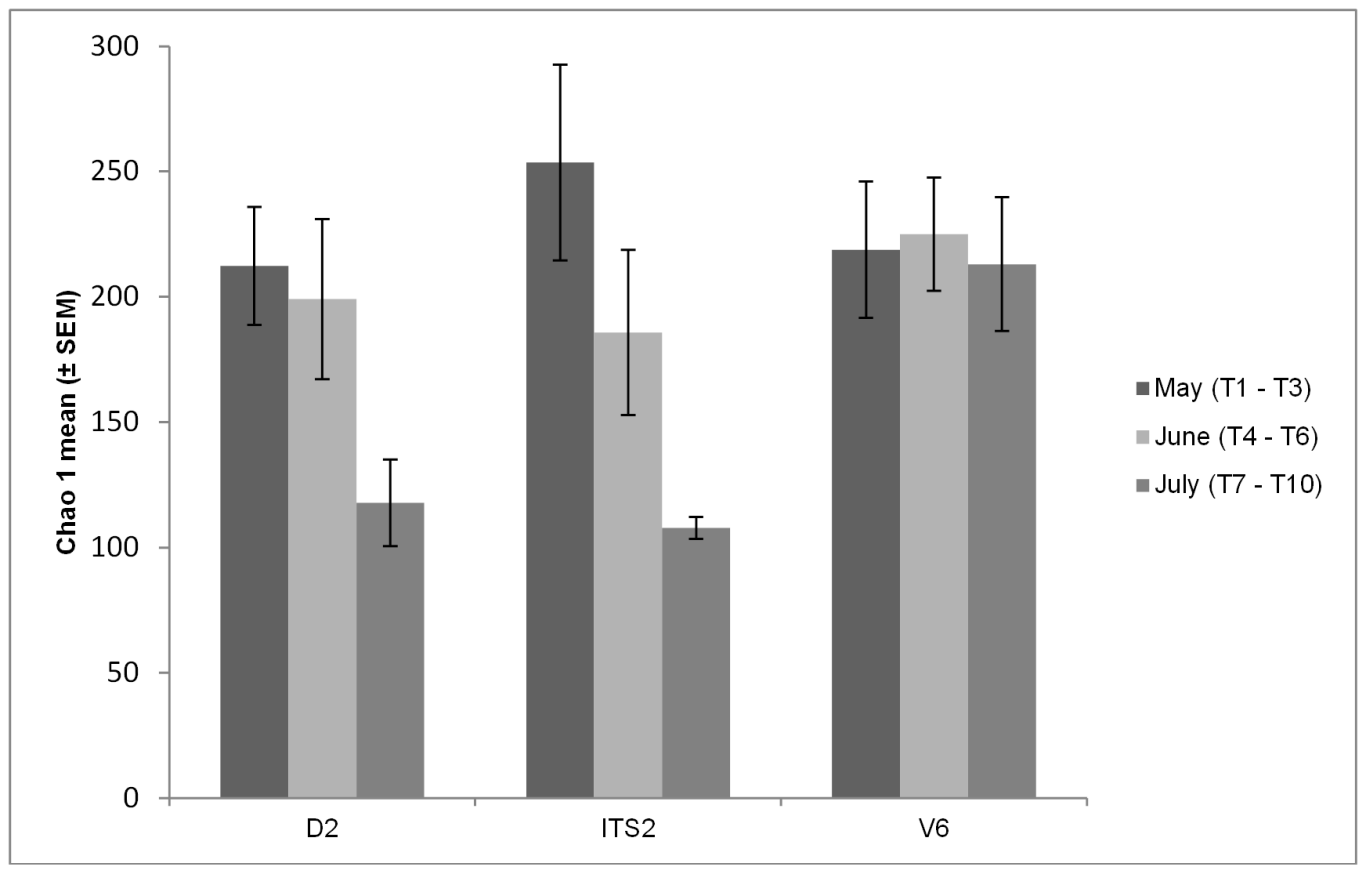
Biodiversity dynamics associated with D2, ITS2 and V6 during the vegetative cycle of grapevine. The means of Chao1 index ± SEM are represented in the graph. Significance was assessed with one-way analysis of variance (ANOVA) and p<0.05 was set as statistic significant level. No significant differences were obtained for D2, ITS2 and V6 regions among May, June and July.

In order to assess to the microbial biodiversity during the plant's vegetative cycle, the Chao1 was determined (Figure 2). Interestingly, the Chao1 varied during the vegetative cycle of grapevine and the sequencing of ITS2 regions exposed a higher biodiversity at May and a lower biodiversity at July when compared with D2 and V6 regions.

### Microbial community composition

The eukaryotic microbiome of the grapevine leaves was mainly characterized by a high amount of microorganisms from Early diverging fungal lineages (27.9%), Ascomycota phylum (26.3%) and Basidiomycota (16.9%), and at much lower abundances we have also identified microorganisms from Chytridiomycota, Blastocladiomycota and Rozella phyla, which all summed up represent 4.3% of the microbial population. The unknown eukaryotic sequences corresponded to 24.7%, meaning that these sequences were not assigned to any microorganism during the BLAST process (Figure S3a).

Again, our results also reinforce the need for simultaneously sequencing of both eukaryotic regions which was crucial because their discriminating power is rather different, where some organisms are only identified by one of these regions (Figure S4).

Regarding the microbial community, the grapevine showed a dominance of microorganisms that belonged to early diverging fungal lineages namely, *Rhizopus*, *Mucor* and the entomopathogens *Zoophthora* and *Pandora*. Among *Rhizopus* and *Pandora*, these microorganisms were very dynamic along the vegetative cycle and showed to have a higher dominance on July. *Rhizopus* is responsible for the sour rot of grapes and for post-harvest diseases in close association with others as *Penicillium*, *Alternaria* or *Diplodia* [26], [27]. The *Mucor* population decreased along the vegetative cycle and, as *Rhizopus*, is an important genera associated with post-harvest diseases of table grapes [28]. Finally, the entomopathogens *Zoophthora radicans* and *Pandora neoaphidis* are insect-pathogenic fungi that infect and kill a variety of insects, including pests [29].

Indeed, some of these early diverging fungi lineages are known to affect the functional insect biodiversity, rather than to impact directly on the physiology of grapevine. For this reason, from now on we will focus on microorganisms belonging to the Ascomycota and Basidiomycota phyla.

**Figure 3:**
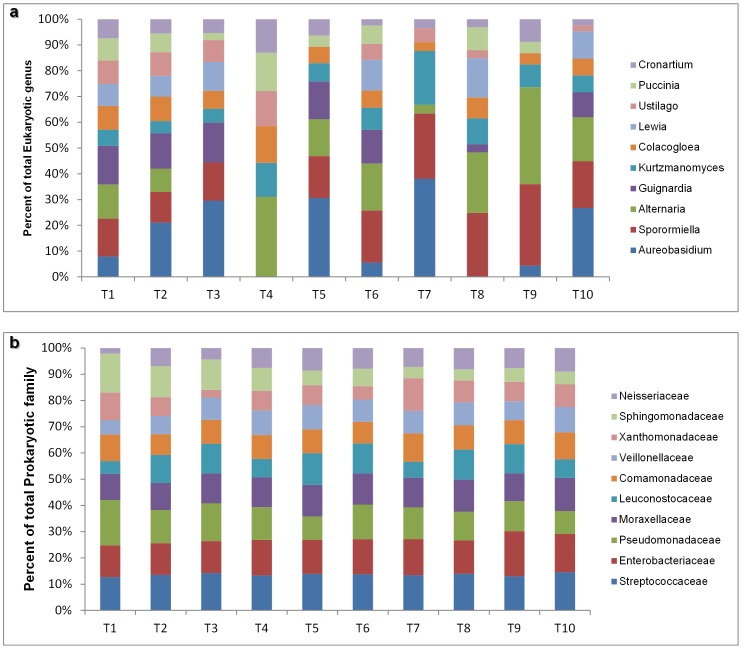
Eukaryotic (a) and prokaryotic (b) microbial community distribution from T1 to T10. Relative abundance of the 10 most abundant eukaryotic and prokaryotic microorganisms through the genus and family analysis, respectively.

Of these, the most dominant genera were *Aureobasidium*, *Sporormiella* and *Alternaria* from Ascomycota phylum (Figure 3a) and the phytopathogen *Guignardia*, which had higher abundances at T1, T2, T3, T5 and T6. At lower abundance, we have identified other genera as *Kurtzmanomyces*, *Colacogloea*, *Lewia*, *Ustilago*, *Puccinia* and *Cronartium*. The eukaryotic community of T1 was the most complex and biodiverse of all samples and, interestingly, such biodiversity consistently decreased during the vegetative cycle. As mentioned above, *Aureobasidium* was dominant, which is in agreement with previously published studies that reported these species as the most abundant in similar eukaryotic communities [30], [31] .

The dominant phylum among prokaryotic community was Proteobacteria with 31.2% and the Firmicutes with 29.4%. The least abundant phylum was Actinobacteria with 19.4% (Figure S3b). At the class level, the microbial communities were mostly characterized by Gammaproteobacteria (18.8%), Bacilli (18.1%), Betaproteobacteria (12.6%), Actinobacteria (12.1%), Alphaproteobacteria (11.2%), Negativicutes (9.9%), unknown microorganisms (8.7%) and a minor abundance of other class bacterial which all summed up represents 8.6% (Figure S3c).

Bacterial community (Figure 3b) was mostly dominated by Streptococcaceae, Enterobacteriaceae, Pseudomonadaceae and Moraxellaceae families followed by Leuconostocaceae, Comamonadaceae, Veillonellaceae, Xanthomonadaceae, Sphingomonadaceae and Neisseriaceae

Although the microbial community seemed to be similar from T1 to T10, the relative abundances varied during the vegetative cycle. For example while T1 was characterized by the major abundance of Pseudomonadaceae and Sphingomonadaceae, T10 was characterized by the dominance of Streptococcaceae and Enterobacteriaceae.

**Figure 4:**
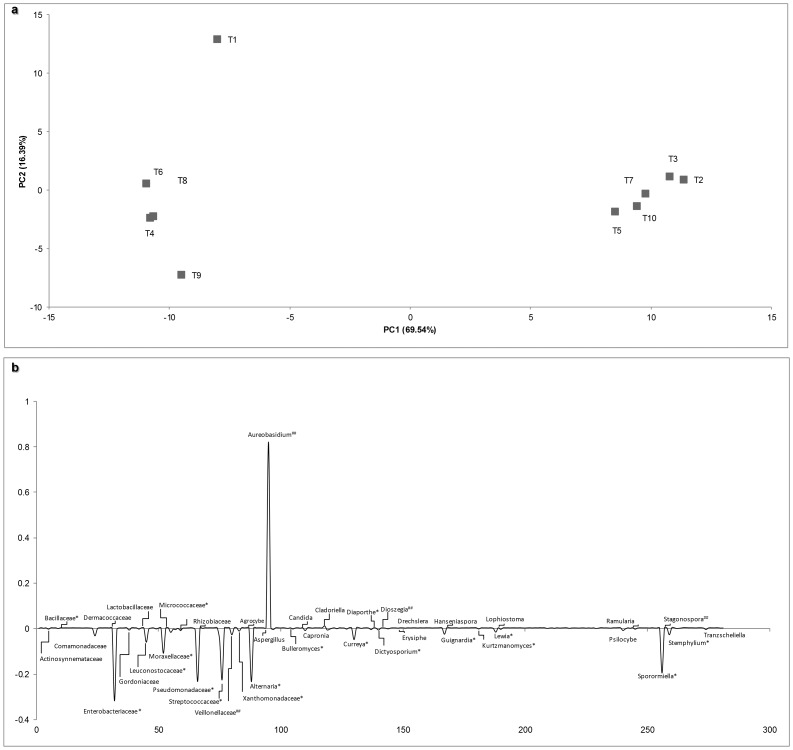
PCA biplot diagram and loading plot of microbial community across sampling time (T1–T10), based on sequence abundance of eukaryotic genus and bacterial family. (a) The PCA diagram is shown and the percentages of data variability explanation are shown in the upper of X and Y axis and more than 85% of the variability in the data is accounted. (b) Loading plot of principal component 1 showing variables that explain variability across eukaryotic genus and prokaryotic family during the vegetative cycle of grapevine (T1–T10). The significant differences were observed in Metastats for eukaryotic genus or prokaryotic families and are represented with asterisk (*) and microbial community that are identified with ## are considered false discovery rate.

To analyze the dynamics and relationships among the entire microbial communities (eukaryotic and prokaryotic) present from T1 to T10, a Principal Component Analysis (PCA) was carried out. Figure 4a shows that this separated samples into twogroups, in terms of similarity degree. The first cluster grouped the sampling times T4, T6 and T8, which corresponded to those samples collected after chemical treatment, whereas the second cluster groups T2, T3, T5, T7 and T10, which were collected both before (T3,T5, T7) and after chemical treatments (T2, T10), and have no correlation with chemical treatment or collection time. Indeed, the separation into these 2 clusters is mainly explained by the presence of Enterobacteriaceae, Pseudomonadaceae, Streptococcaceae, *Alternaria* and *Sporormiella*, in the first cluster, and the presence of *Aureobasidium* in the second cluster (Figure 4b). Furthermore T1, which does not belong to any of the clusters formed by the hierarchical clustering based on a Pearson correlation matrixes, is mainly characterized by the major abundances of Guignardia, a phytopathogens, and Pseudomonadaceae.

### Impact of the chemical treatments on microbial community

**Figure 5:**
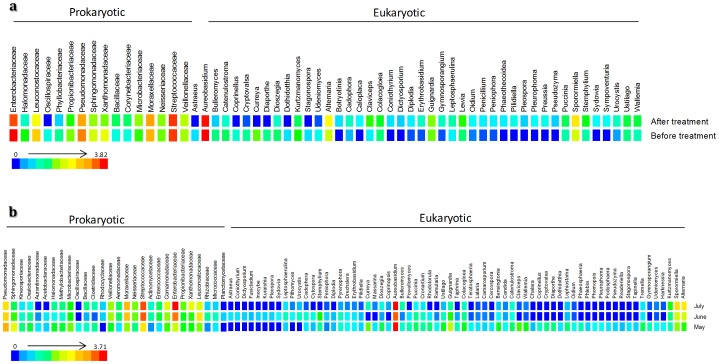
Variation on the abundance of the significant eukaryotic genus and prokaryotic family (p<0.05) as affected by chemical treatment (a) and grapevine vegetative cycle (b). The variation on population during the grapevine vegetative cycle could be interpreted through the variation of the square's color where the red square indicates a higher number of reads and a blue square denotes a reduce number of reads of a specific microorganism. Only the significant population is shown (p<0.05).

The chemical treatments affected the vineyard's microbial population and the comparison among microbial community using Metastats [24] revealed differences between communities (ρ<0.05) (Figure 5a). In general, chemical treatments had a negative impact on the balance between phytopathogens and phytoprotectors in the *V. vinifera* microbiome (Figure S5), and a significant decrease on population was observed after the first treatment on May (Figure 5b), when there was the highest microbial biodiversity in the vineyard. Considering the eukaryotic community (ρ<0.05) we found significant differences in the populations of *Alternaria, Bulleromyces, Claviceps, Cryptovalsa, Diaporthe, Guignardia, Lewia, Pleurophoma, Puccinia, Sporormiella, Stemphylium, Sydowia* and *Ustilago* (Figure 5a; Table S2).

*Aureobasidium*, the most abundant eukaryotic genus, showed a relative abundance of 7.1% and 4.1% before and after chemical treatments, respectively. *Sporormiella* (6.1 and 5.1%) and the phytopathogens *Alternaria* (3.9 and 4.2%) and *Guignardia* (3.3 and 3.0%) were also abundant (Figure S6a). Interestingly, we have identified a negative correlation between *Aureobasidium* and *Alternaria*: when *Aureobasidium* is present, *Alternaria* is reminiscent and vice-versa. *Alternaria* is mainly present after the chemical treatment when *Aureobasidium* is less abundant, which suggest that *Aureobasidium* have a protector effect on plant and its abundance on microbial community is clearly affected by chemical control. Among the rare eukaryotic genera, we found *Filobasidiella*, *Diaporthe* (the teleomorph of *Phomopsis viticola*), *Cryptovalsa*, *Stemphylium*, *Candida*, *Phomopsis*, *Botryotinia*, *Dothichiza*, *Bulleromyces* and *Dioszegia*. Interestingly, among this low abundance microorganisms, we have observed *Botryosphaeria dothidea* (0.23%), which is a phytopathogen associated with grapevine trunk disease that causes the decline of grapevine, limiting vineyard longevity and productivity [32].

In our analysis, *Saccharomyces*, *Hanseniaspora* and *Metschnikowia* were also identified in leaves at T3, T6 and T8, though at low levels (<1%). Our data demonstrated that despite being at low levels, these microorganisms are natural colonizers of the vine, even before the appearance of grape fruits and after chemical treatments.

When chemical control with the active element sulfur is applied (Table S3), a drastic impact on the yeast flora is observed [33], and this explains the observed decrease of Aureobasidium, Rhodotorula or Candida by the end of May. In general, the whole yeast community was affected by these chemical treatments (Figure 5b). Furthermore, the application of the chemical treatments supplemented with folpet (applications between T3–T4, T5–T6 and T7–T8) showed an impact mostly in Aureobasidium during May and June which is in accordance with the study of Cabras et al. [34]. Folpet is generally used to control downy mildew, powdery mildew and grey mold infections which are the most devastating grapevine diseases [35]. In our study we did not find the most damaging phytopathogens of grapevine namely, downy mildew (*Plasmopara viticola*) and powdery mildew (*Uncinula necator*) (syn. *Erysiphe necator*) [36], [37] which might be explained by the constant chemical control of these phytopathogens (Table S3).

Our experimental vineyard was also subjected to the treatments against black rot disease (*Guignardia bidwelli*) and phomopsis cane and leaf spot (*Phomopsis viticola*). As expected, after the chemical control the abundance of these phytopathogens decreased dramatically (Figure 5; table S1). Interestingly, only a chemical treatment was applied against *G. bidwelli* and we have observed that during the vegetative cycle new infections have emerged on the vineyard (Figure S5b).

Concerning the prokaryotic population, we found significant differences across Bacillaceae, Corynebacteriaceae, Enterobacteriaceae, Halomonadaceae, Leuconostocaceae, Microbacteriaceae, Moraxellaceae, Propionibacteriacea, Pseudomonadaceae, Sphingomonadaceae, Streptococcaceae and Xanthomonadaceae families (ρ<0.05) (Figure 5a; Table S2).

The most abundant family was Enterobacteriaceae, with a relative abundance of 7.1% and 7.0% before and after chemical treatments, respectively (Figure S6b). Also, Streptococcaceae (7.0% and 7.3%), Pseudomonadaceae (6.5% and 6.1%), Moraxellaceae (5.8% and 6.2%) and Comamonadaceae (5.0% and 4.7%), were among the most abundant before and after chemical treatments of the vineyards, respectively. Other families, as Methylobacteriaceae, Oxalobacteraceae, Nocardioidaceae, Rhodobacteraceae or Bacillaceae were also detected, though with relative abundances below 2%, and were considered as rare microorganisms. In general, the applied chemicals affected the bacterial community and a decreased on the relative abundance was observed after chemical treatments for Enterobacteriaceae, Pseudomonadaceae, Comamonadaceae or Xanthomonadaceae families.

In the grapevine bacterial population, both lactic acid bacteria (LAB) and acetic acid bacteria (AAB) were identified among other bacterial groups. In wine production LAB, especially *Oenococcus oeni, Lactobacillus, Pediococcus* and *Leuconostoc* are of outmost importance because of the malolactic fermentation. In our samples, the identified LAB belonged to Carnobacteriaceae, Enterococcaceae, Leuconostocaceae and Streptococcaceae families. From the Carnobacteriaceae it was observed *Trichoccus* and *Atopostipes* genus and from the Enterococcaceae family *Enterococcus* (mainly *E. italicus*) was also observed. From the Leuconostocaceae we observed *Leuconostoc* (*L. citreum* and *L. fallax* species) and *Weisella* (*W. confuse*) and, finally, from the Streptococcaceae family, *Streptococcus* and *Lactococcus* were identified. However, most of these bacteria are not associated with the winemaking process. We have also detected microorganisms from the Lactobacillaceae family mainly, *Lactobacillus salivarius*, though this population was rare (table S1). Like previous reports on biodiversity of grapes, we did not identify *O. oeni* in grape leaves [38], [39]. Overall, our data show an increase of LAB from May to July (Figure 5b).

Regarding the AAB, we have detected in our samples microorganisms from the Acetobacteraceae family, mainly *Acidisoma*, *Gluconacetobacter* and *Roseomonas* genus. Remarkably, the AAB was predominantly present on May and June in a positive correlation with the presence of *Botryotinia*. This finding reinforces the positive correlation between AAB and *Botrytis* infection in the vineyards [40], [41].

### Distribution and interactions of microbial community across vegetative cycle

The eukaryotic microbial community was very dynamic during the vegetative cycle (Figure 5b) and presented significant alterations in its structure (ρ<0.05). Among phytopathogens, differences were found in *Guignardia*, *Diaporthe* or *Phomopsis* and between phytoprotectors differences were found in *Aureobasidium* and *Rhodotorula* (table S4).

Concerning the 10 most abundant eukaryotic communities on May, June and July (Figure S7a, 7b and 7c), we observed that on these months the most abundant microorganisms were *Aureobasidium* and *Alternaria*. Furthermore, in May *Guignardia* was the most abundant phytopathogen though *Bensingtonia*, *Claviceps*, *Ustilago*, *Alternaria or Curreya* were also present. On June, *Sporormiella* and *Alternaria* showed an increase and a decrease of *Guignardia* from May to July was detected. Then, on July *Alternaria*, *Aureobasidium* and *Sporormiella* were the most abundant microorganisms and an increase of *Alternaria*, *Aureobasidium* and *Sporormiella* was observed.

Prokaryotic population was also very dynamic and significant differences (ρ<0.05) across the microorganisms as Enterobacteriaceae, Pseudomonadaceae, Streptococcaceae, Sphingomonadaceae, Moraxellaceae, Leuconostocaceae were observed (Figure 5b and table S4).

Of the 10 most abundant prokaryotic communities (Figure S7d, 7e and 7f) we observed that on May Pseudomonadaceae, Streptococcaceae, Sphingomonadaceae and Enterobacteriaceae dominated the microbial consortia. On June, Streptococcaceae was the most abundant family followed by microorganisms from the Enterobacteriaceae, Moraxellaceae and Pseudomonadaceae families. Finally, on July, the most abundant families were Enterobacteriaceae and Streptococcaceae.

## Discussion

In this work we have uncovered the microbial biodiversity of grapevine leaves through metagenomic approaches and the interpretation of microbial communities showed to be essential to understand the balance of phytopathogens and beneficial microorganisms. Such understanding could represent a crucial step for the development of environmental friendly strategies for plant protection and grape production.

### Grapevine phytoprotectors vs. phytopathogens

The abundant eukaryotes and prokaryotes identified belonged to the *Aureobasidium* genus and Enterobacteriaceae family, respectively, which is in agreement with previous published studies [39]. These microorganisms play an important role in the microbial consortium of vineyards and grapes and are thought to be beneficial. They have specific modes of action as induction of host resistance and production of glucanases, chitinases and proteases, which makes them excellent antagonists and also beneficial microorganisms [42].

According to our data, the prevalence of *Aureobasidium* genus is due to the presence of *A. pullulans*. Previous data related that *A. pullulans*, *Epicoccum nigrum*, *Rhodotorula* and *Candida* dominate the consortia of grapes and together are the most abundant anti-phytopathogen microorganisms [31], [43]–[45]. Further, published data also refers that *A. pullulans* has antagonistic activity against moulds, namely *Botrytis* and certain bacterial as *Bacillus* [31], which may explain the lower prevalence of *Bacillus* genus in our results.

In our samples we have also detected *Bulleromyces*, namely *B. albus* and *Dioszegia* spp. The former is referred as a beneficial microorganism with antagonistic activities and with the capacity to produce extracellular polysaccharides [46] and the latter was described to be associated with arbuscular mycorrhizal fungi, revealing a beneficial action [47], [48]. Beyond these, also the yeasts *Sporobolomyces* and *Candida*, have been reported to have antifungal effects [49]. Those yeasts do not have any enological interest and are described as natural inhabitants of the vineyard [9].

Interestingly, a wide diversity of eukaryotic phytopathogens was present in the analysed leaf samples amongst which *Rhizopus*, *Lewia*, *Alternaria*, *Diaporthe*, *Phomopsis*, *Cryptovalsa*, *Stemphylium*, *Ustilago* and *Botryotinia*. Some of these phytopathogens as *Phomopsis* (*Phomopsis* type 2), *Cryptovalsa* (*C.ampelina*) and *Botryotinia* are commonly associated with diseases in the vineyard [50], [51]. Other microorganisms as *Lewia*, *Alternaria*, namely, *A. solani* and *Stemphylium*, (*S. solani*) have been reported as phytopathogens of different crop cultures as wheat, sorghum, pistachio, potatoes and tomatoes [52], [53].

It is worthwhile to notice the emergence of *Guignardia* (*G. bidwelli*) on grapevines, which was one of the most abundant phytopathogens in our samples, has been recently detected in some Portuguese vineyards and it was noticed for the first time on Bairrada appellation during the 2006 vine campaign [54]. *G. bidwelli* causes the black rot and, according to the severity of the disease, the qualitative and quantitative performance of the vineyard could be drastically affected. To date, *G. bidwelli* is restricted to some viticulture regions and in Portugal their occurrence is higher at Bairrada and Alentejo appellations [55].

Among prokaryotic consortia, we have also identified potential antagonistic microorganisms. According to previous studies, the most well-known and reported bacterial antagonists are species of *Pseudomonas* (*Pseudomonadaceae* family), *Burkholderia* (*Burkholderiaceae*), *Bacillus* (*Bacillaceae*), *Serratia* and *Pantoea* (*Enterobacteriaceae*) and *Actinomycetes* (*Actinomycetaceae*) [56], [57]. It is well documented that bacterial strains belonging to *Streptococcus* (*Streptococcaceae* family) also display antagonistic activity against fungal pathogens [56]. In our samples, all these bacterial were detected although *Burkholderiaceae* where not abundant.

Additionally, the LAB found in our study are referred to be widespread in fermentable materials and, because of their potential of acidification, they prevent the growth of phytopathogenic microorganisms and have inhibitory effects on yeasts [38], [58].

Furthermore, a wide diversity of bacterial microorganisms co-habit with grapevine but do not cause adverse effects, with exception of *Pseudomonas syringae* and *Xanthomonas campestris* pv. *viticola* that are described as phytopathogens but were not detected on our analysis. However, and with exception of some bacteria that have impact in wine production, most of the bacterial population that we found on grapevine are not integrated in the wine microbial consortium and do not influence wine quality [59].

### Microorganism's modulation in the vineyards

The vineyard's microbial population showed to be very dynamic across the vegetative cycle and a high biodiversity was unveiled. Nevertheless, a sharp decline in eukaryotic biodiversity was observed during the grapevine ripening which can be explained by spatial and temporal fluctuations, biotic and abiotic factors or other conditional factors as chemical treatments [59]. Beyond these, viticulture practices, grapevine varieties, age of vines, grapevine ripening and vectors are all known to influence the microbial ecosystem, microbial dispersal and even microbial performance [45], [60]. Nevertheless, microbial community is ubiquitous and some of them are responsible to maintain the ecosystem function.

Despite the unveiled high biodiversity, the observed balance between the different microorganisms and the dynamics across grapevine ripening, there is still a large gap in the knowledge of the functional diversity and significance of microbial community-plants interaction on grapevine. In fact, the co-existence of different microbial population generates competition for the nutrients, different interactions are established and enzymatic compounds are produced. Indeed, the latter could have a toxic effect on other species, and thus have antifungal properties [43], [58], 61.

The deep analysis of the microbial consortia revealed statistically significant differences in eukaryotic and prokaryotic diversity within chemical treatments and during the vegetative cycle, which will have a direct and indirect effect on grapevine community composition. Indeed, among eukaryotic population the early diverging fungal lineages and Ascomycota phyla and the prokaryotic Proteobacteria showed higher abundances in vineyards.

Altogether, this work reinforced the importance of studying the natural biodiversity of grapevine and highlighted the need of a more detailed study of the microbial interactions on plant. Furthermore, the grapevine microbial consortia showed to contain both beneficial and phytopathogenic microorganisms which will have a significant influence on the vine performance and also on the wine quality. Our data contribute to the characterization of the biodiversity of grapevines and to the analysis of biomarkers with the potential to unveil the plant health status.

## Supporting Information

Figure S1 **Vineyard chosen for study and chemical treatments calendar.** (a) Sample collection was done in the 5 vines, throughout the 10 time points (T1 to T10). To ensure reliable results, all samples were collected from the same vines. (b)The time intervals of samples collection was defined according to the chemical treatments calendar, over the 3 months of trial. The leaves were collected before and closed to the chemical treatment application (green plot) and after the chemical treatment (red plot).
(PNG)

Figure S2 **Venn diagram showing the observed species for ITS2 and D2 region and common species.** The number of reads and the OTUs obtained are showed for both regions. Taxonomic classification was defined by 97% of sequence similarity. To determine which region of the 26S rDNA would be most suited for the metagenomic analysis of eukaryotic microorganisms, a preliminary test was carried out where within the same sample we have targeted both ITS2 and D2 regions. After this analysis, we have obtained 123 observed microorganisms for ITS2 and 121 for D2 region although, just 41 microorganisms were common to both regions.
(TIF)

Figure S3 **Microbial community distribution over the vegetative cycle of grapevine.** Relative abundance of the eukaryotic microorganisms (a) that were mostly characterized by Early diverging fungi and Ascomycota phyla. The prokaryotic community (b) was characterized by Proteobacteria and Firmicutes and at the class level (c) by Gammaproteobacteria and Bacilli.
(TIF)

Figure S4 **Relative abundance of the number of reads during the grapevine vegetative cycle of the eukaryotic population.** Detailed description of the relative abundance of D2 (a) and ITS2 sequences (b) during sampling collection. A deep analysis of D2 region showed that early diverging fungal lineages were only identified by D2 sequencing and also this region identified predominantly microorganisms designated as others. According to ITS2, the major relative abundance was of Ascomycota and unknown microorganisms.
(TIF)

Figure S5 **Effect of chemical treatment application on specific microorganisms.** The balance of microbial community is affected by chemical treatments and a decrease of both phytopathogens (a,b) and phytoprotectors (c,d) is observed. The chemical control was applied between the intervals T1 and T2, T3 and T4, T5 and T6, T7 and T8, T9 and T10. The arrows indicate the application of chemical treatments with known direct effect on presented microorganisms.
(TIF)

Figure S6 **Relative abundance (%) of eukaryotic genus and prokaryotic family before and after the chemical treatment.** The most abundant phylogenetic groups (>2%) of eukaryotic genus (a) and prokaryotic family (b).
(TIF)

Figure S7 **Microbial Composition of May (a and d), June (b and e) and July (c and f).** Relative abundance of the 10 most abundant eukaryotic (a, b and c) and prokaryotic (d, e and f) microorganisms for each month, through the genus and family analysis, respectively.
(TIF)

Table S1 Species observed on eukaryotic and prokaryotic community. Distribution of the microbial microorganisms identified both eukaryotic and prokaryotic, during the vegetative cycle of grapevine. For each microorganism, a consensus ID, number of reads, alignment lenght, alignment start, score and the respective e-value for the blast are shown.
(XLSX)

Table S2 Impact of chemical treatments application on eukaryotic and prokaryotic community. Application of Metastat to compare and to detect differences between microbial communities (both eukaryotic and prokaryotic) with chemical treatment application.
(XLSX)

Table S3 Active elements of chemical treatments. Chemical treatments calendar with their respective active elements. The interval of application of such chemical treatment is also shown.
(XLSX)

Table S4 Distribution and interactions of microbial community across vegetative cycle. Application of Metastat to compare and to detect differences between microbial communities (both eukaryotic and prokaryotic) during the vegetative cycle of grapevine.
(XLSX)
