# Supplementary figures and images for: Unravelling the Diversity of Grapevine Microbiome
Source: PLoS One. 2014 Jan 16;9(1):e85622. doi: 10.1371/journal.pone.0085622 (PMC3894198; doi:10.1371/journal.pone.0085622)

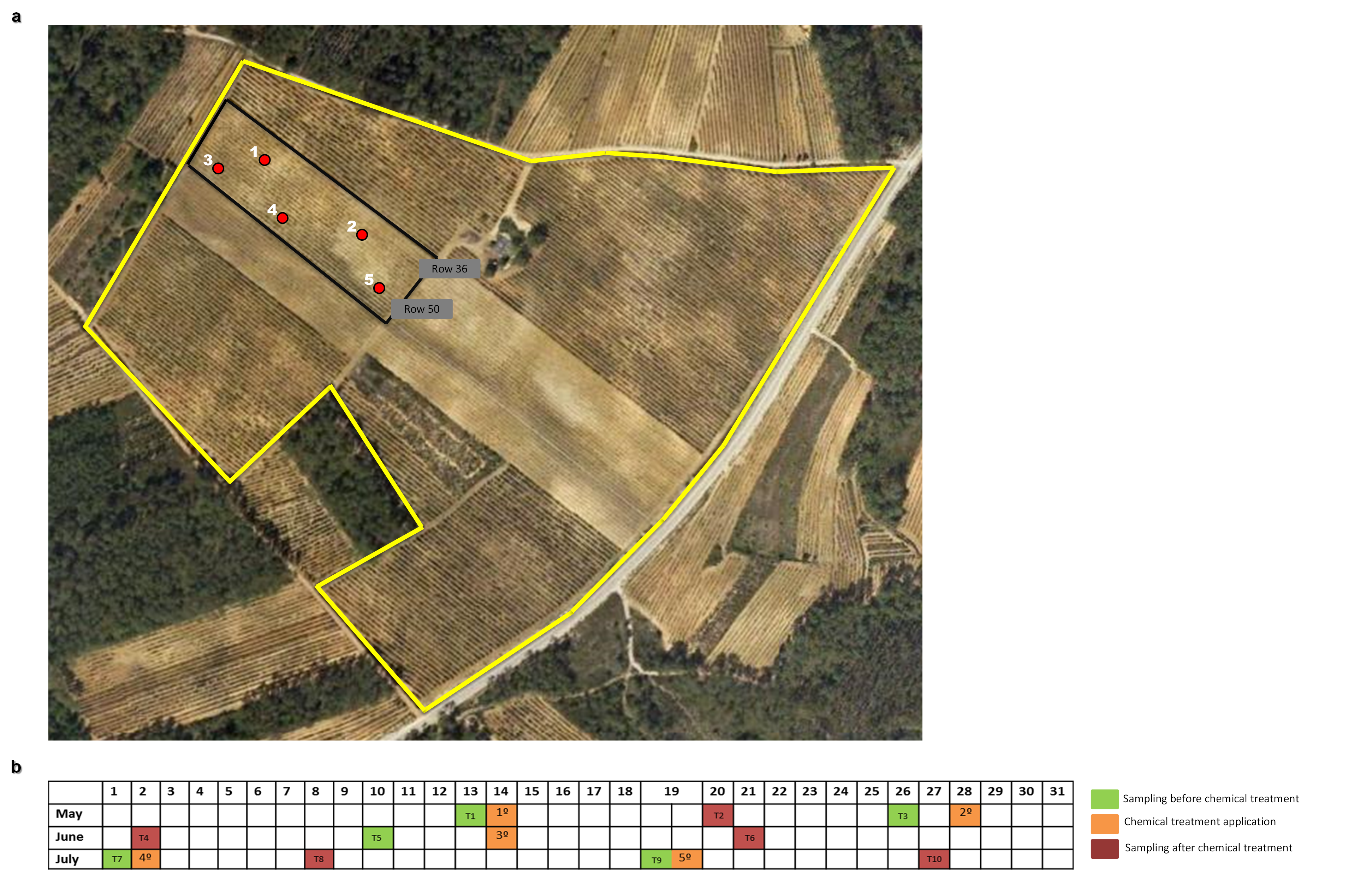

Supplement: Figure S1 — Vineyard chosen for study and chemical treatments calendar. (a) Sample collection was done in the 5 vines, throughout the 10 time points (T1 to T10). To ensure reliable results, all samples were collected from the same vines. (b)The time intervals of samples collection was defined according to the chemical treatments calendar, over the 3 months of trial. The leaves were collected before and closed to the chemical treatment application (green plot) and after the chemical treatment (red plot). (PNG) [file pone.0085622.s001.png]

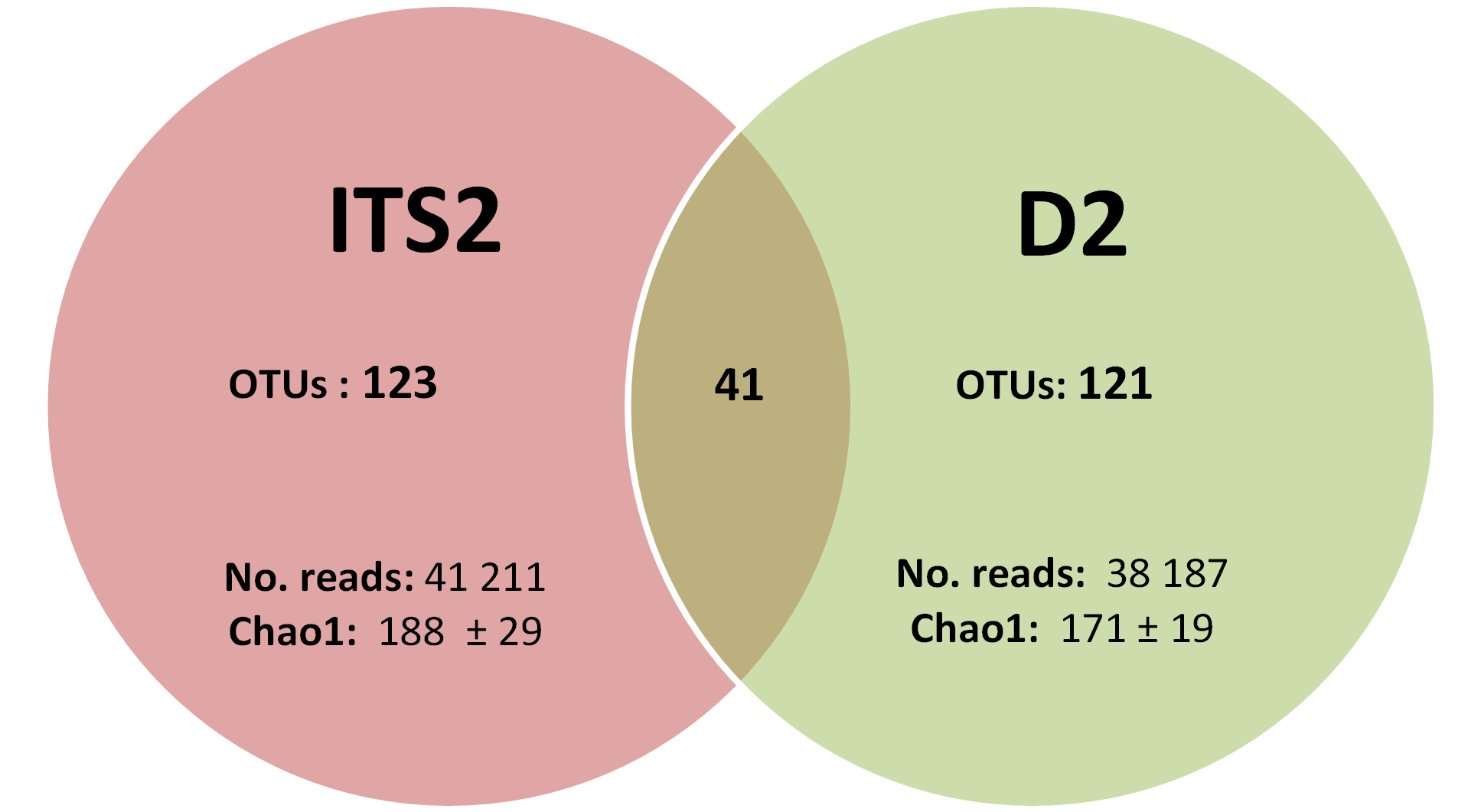

Supplement: Figure S2 — Venn diagram showing the observed species for ITS2 and D2 region and common species. The number of reads and the OTUs obtained are showed for both regions. Taxonomic classification was defined by 97% of sequence similarity. To determine which region of the 26S rDNA would be most suited for the metagenomic analysis of eukaryotic microorganisms, a preliminary test was carried out where within the same sample we have targeted both ITS2 and D2 regions. After this analysis, we have obtained 123 observed microorganisms for ITS2 and 121 for D2 region although, just 41 microorganisms were common to both regions. (TIF) [file pone.0085622.s002.tif]

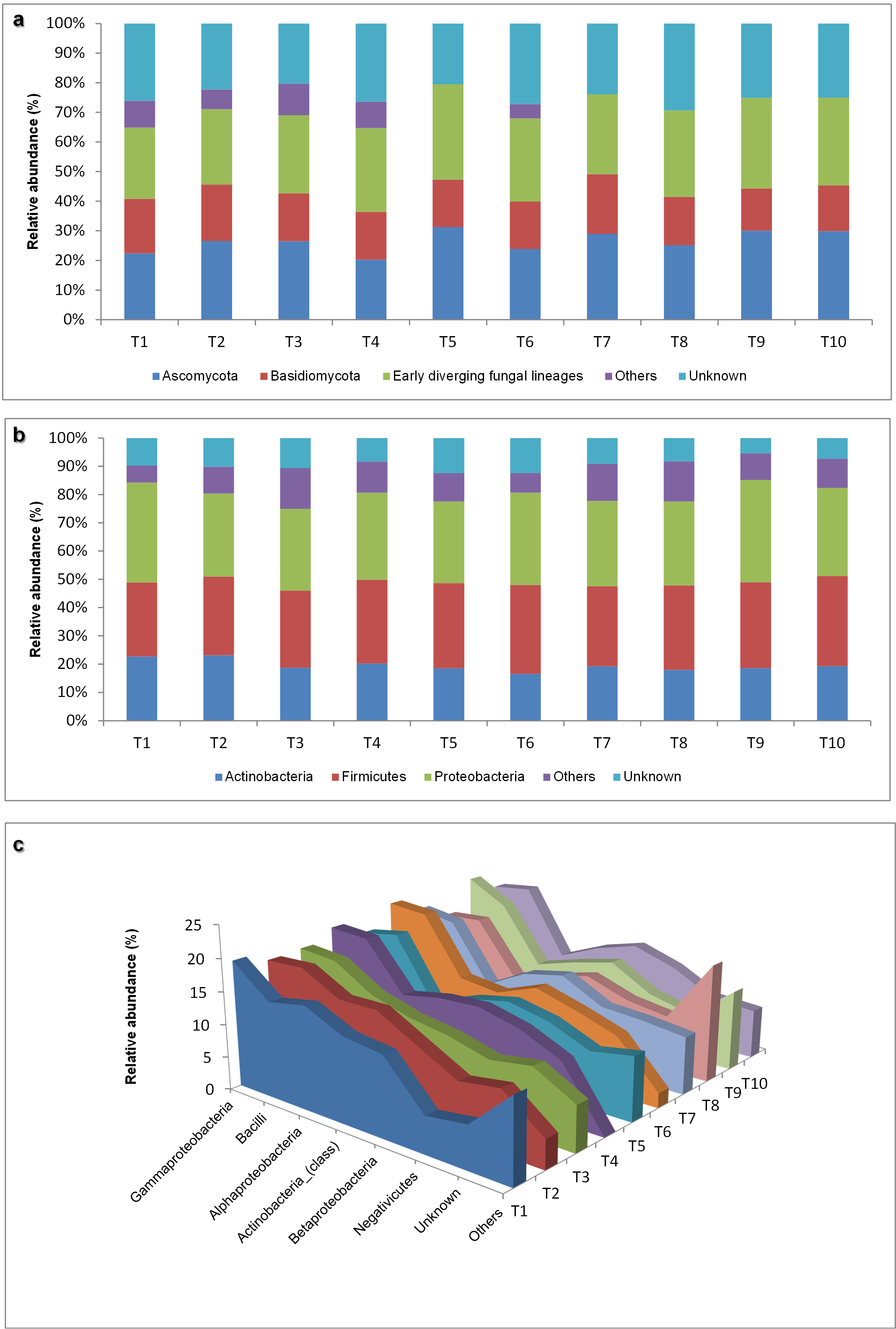

Supplement: Figure S3 — Microbial community distribution over the vegetative cycle of grapevine. Relative abundance of the eukaryotic microorganisms (a) that were mostly characterized by Early diverging fungi and Ascomycota phyla. The prokaryotic community (b) was characterized by Proteobacteria and Firmicutes and at the class level (c) by Gammaproteobacteria and Bacilli. (TIF) [file pone.0085622.s003.tif]

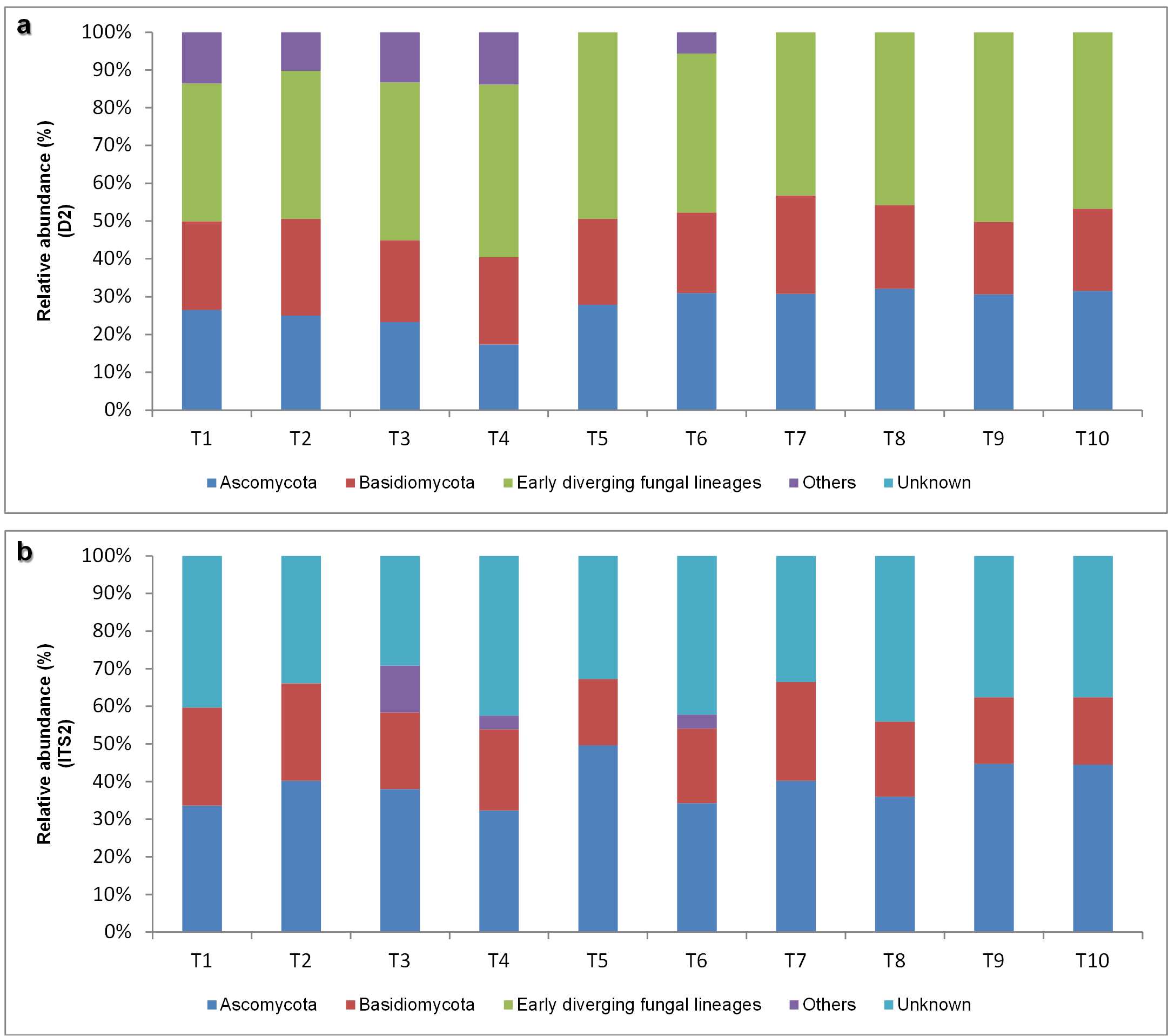

Supplement: Figure S4 — Relative abundance of the number of reads during the grapevine vegetative cycle of the eukaryotic population. Detailed description of the relative abundance of D2 (a) and ITS2 sequences (b) during sampling collection. A deep analysis of D2 region showed that early diverging fungal lineages were only identified by D2 sequencing and also this region identified predominantly microorganisms designated as others. According to ITS2, the major relative abundance was of Ascomycota and unknown microorganisms. (TIF) [file pone.0085622.s004.tif]

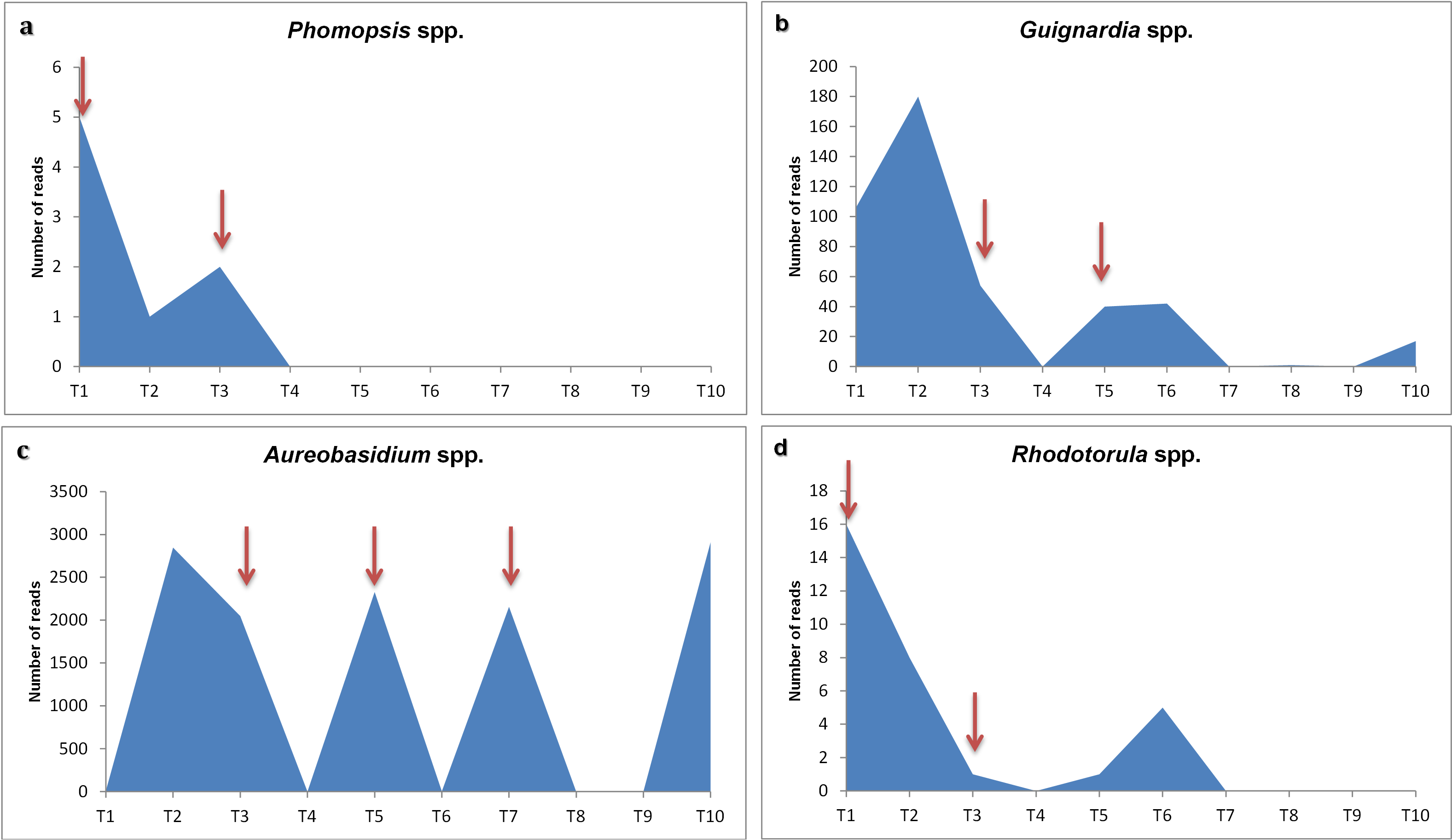

Supplement: Figure S5 — Effect of chemical treatment application on specific microorganisms. The balance of microbial community is affected by chemical treatments and a decrease of both phytopathogens (a,b) and phytoprotectors (c,d) is observed. The chemical control was applied between the intervals T1 and T2, T3 and T4, T5 and T6, T7 and T8, T9 and T10. The arrows indicate the application of chemical treatments with known direct effect on presented microorganisms. (TIF) [file pone.0085622.s005.tif]

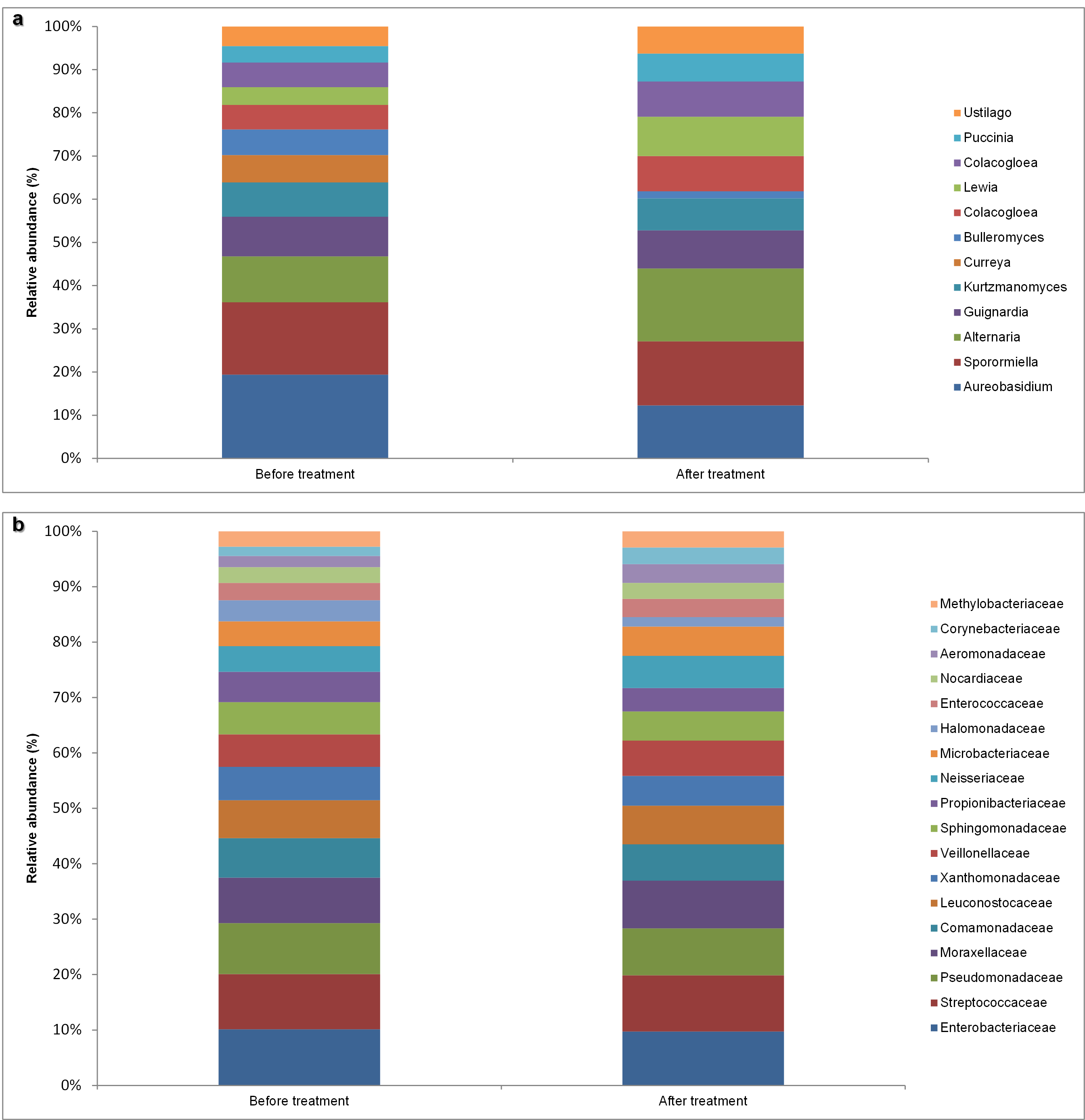

Supplement: Figure S6 — Relative abundance (%) of eukaryotic genus and prokaryotic family before and after the chemical treatment. The most abundant phylogenetic groups (>2%) of eukaryotic genus (a) and prokaryotic family (b). (TIF) [file pone.0085622.s006.tif]

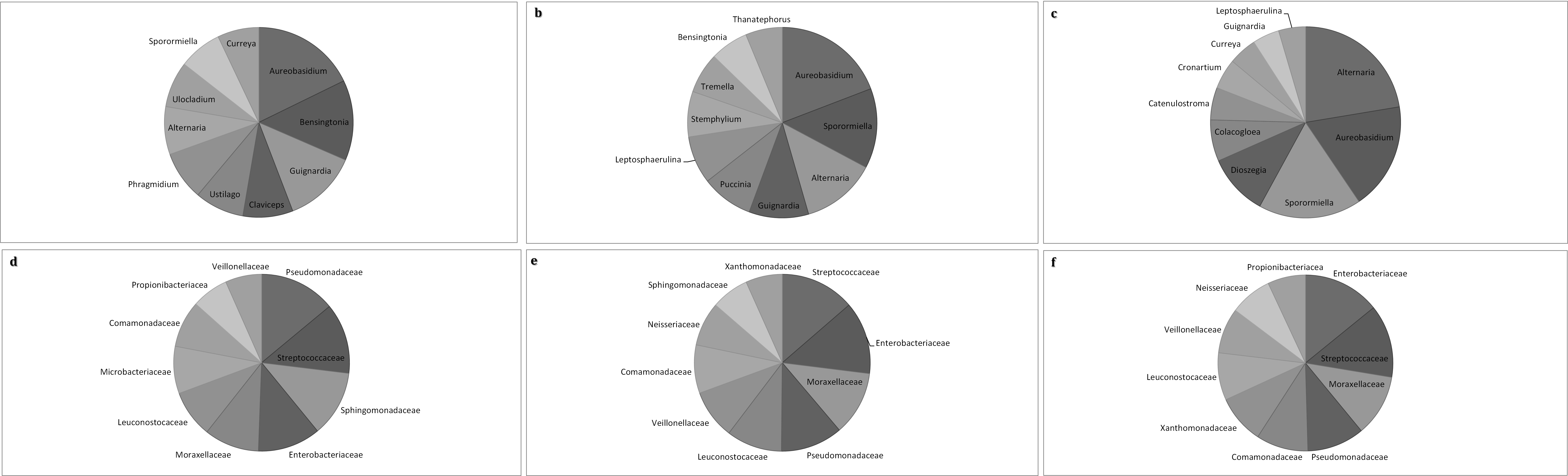

Supplement: Figure S7 — Microbial Composition of May (a and d), June (b and e) and July (c and f). Relative abundance of the 10 most abundant eukaryotic (a, b and c) and prokaryotic (d, e and f) microorganisms for each month, through the genus and family analysis, respectively. (TIF) [file pone.0085622.s007.tif]
